# Molecular and fossil evidence place the origin of cichlid fishes long after Gondwanan rifting

**DOI:** 10.1098/rspb.2013.1733

**Published:** 2013-11-07

**Authors:** Matt Friedman, Benjamin P. Keck, Alex Dornburg, Ron I. Eytan, Christopher H. Martin, C. Darrin Hulsey, Peter C. Wainwright, Thomas J. Near

**Affiliations:** 1 Department of Earth Sciences, University of Oxford, South Parks Road, Oxford OX1 3AN, UK; 2 Department of Ecology and Evolutionary Biology, University of Tennessee, Knoxville, TN 37996, USA; 3 Department of Ecology and Evolutionary Biology and Peabody Museum of Natural History, Yale University, New Haven, CT 06520, USA; 4 Department of Evolution and Ecology, University of California, Davis, CA 95616, USA

**Keywords:** biogeography, dispersal, fossil record, molecular clock, vicariance

## Abstract

Cichlid fishes are a key model system in the study of adaptive radiation, speciation and evolutionary developmental biology. More than 1600 cichlid species inhabit freshwater and marginal marine environments across several southern landmasses. This distributional pattern, combined with parallels between cichlid phylogeny and sequences of Mesozoic continental rifting, has led to the widely accepted hypothesis that cichlids are an ancient group whose major biogeographic patterns arose from Gondwanan vicariance. Although the Early Cretaceous (*ca* 135 Ma) divergence of living cichlids demanded by the vicariance model now represents a key calibration for teleost molecular clocks, this putative split pre-dates the oldest cichlid fossils by nearly 90 Myr. Here, we provide independent palaeontological and relaxed-molecular-clock estimates for the time of cichlid origin that collectively reject the antiquity of the group required by the Gondwanan vicariance scenario. The distribution of cichlid fossil horizons, the age of stratigraphically consistent outgroup lineages to cichlids and relaxed-clock analysis of a DNA sequence dataset consisting of 10 nuclear genes all deliver overlapping estimates for crown cichlid origin centred on the Palaeocene (*ca* 65–57 Ma), substantially post-dating the tectonic fragmentation of Gondwana. Our results provide a revised macroevolutionary time scale for cichlids, imply a role for dispersal in generating the observed geographical distribution of this important model clade and add to a growing debate that questions the dominance of the vicariance paradigm of historical biogeography.

## 1. Introduction

**Figure 1.**
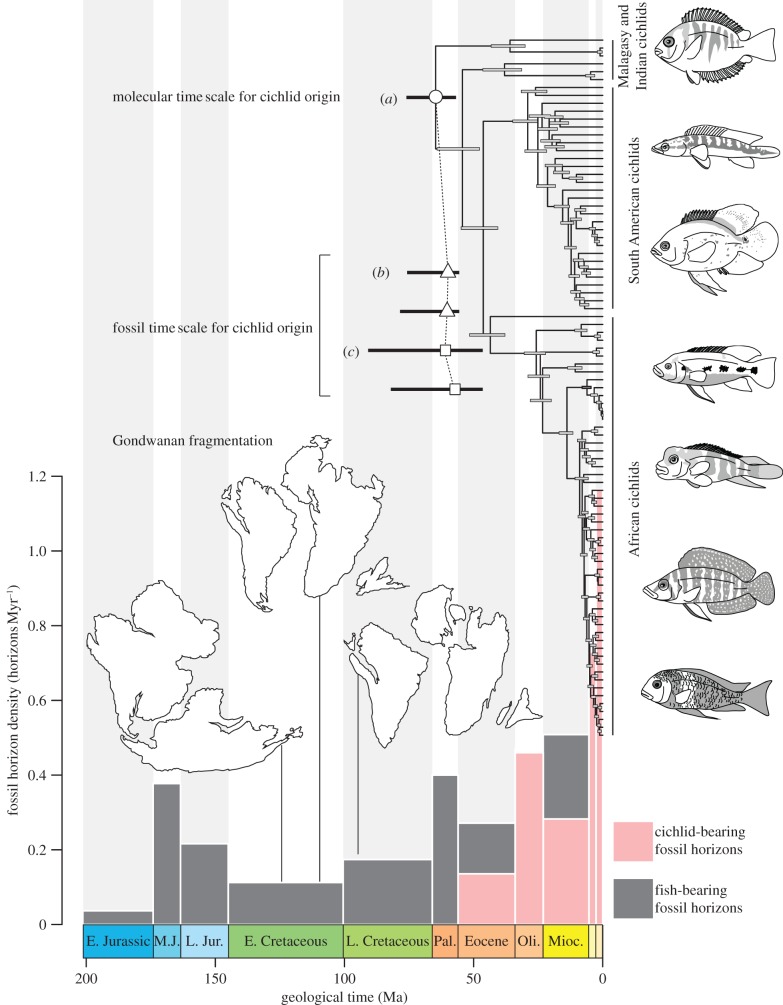
Congruent molecular and palaeontological time scales place the origin of cichlid fishes in the Late Cretaceous–Eocene interval, substantially post-dating Gondwanan rifting. (*a*) Molecular phylogeny for Cichlidae calibrated using fossils belonging to non-cichlid groups (full phylogeny provided in electronic supplementary material, figures S1 and S2). (*b*) Bayesian point estimates and 95% CIs for the timing of cichlid origin based on the distribution of cichlid fossils and the availability of freshwater sedimentary deposits of Triassic–Recent age on Gondwanan landmasses that bear articulated fish remains. The top estimate is derived from the record of landmasses inhabited by extant cichlids, and the bottom estimate is derived from the record of all Gondwanan landmasses. The density of all Gondwanan horizons bearing articulated freshwater fish fossils is indicated by the histogram at the bottom of the figure (densities including disarticulated material are given in electronic supplementary material). Grey bars indicate total horizon density. Pink bars indicate the density of the subset of fossil fish horizons that bear cichlids. (*c*) Bayesian point estimates and 95% CIs for the timing of cichlid origin based on successive fossil outgroups to the clade. The two estimates reflect competing hypotheses for the earliest fossil examples of some outgroups. The top estimate is based on the oldest proposed outgroup ages and the bottom estimate is based on the youngest proposed outgroup ages. Cichlid illustrations, from top to bottom: *Etroplus*, *Crenicichla*, *Astronotus*, *Hemichromis*, *Steatocranus*, *Altolamprologus* and *Tropheus*. Continental arrangements based on palaeogeographic reconstructions by R. Blakey, originally presented in [7].

Cichlid fishes, along with Darwin's finches and Caribbean *Anolis* lizards, represent a key vertebrate model system for understanding the evolutionary assembly of biodiversity [1,2]. Despite the group's prominence in biological research, a consistent macroevolutionary time scale and biogeographic history for cichlids has remained elusive [3–6]. For nearly four decades, the study of deep cichlid evolutionary history has been dominated by vicariance models of biogeography that link the present-day distribution of the group to the tectonic fragmentation of the supercontinent of Gondwana during the mid to late Mesozoic (*ca* 135–90 Ma; figure 1) [8,9]. Continued investigation of cichlid intrarelationships, including phylogenetic analysis of molecular sequence data, has shown congruence between the order of divergences among geographically restricted cichlid clades and proposed sequences of continental break-up [10]. The vicariance hypothesis of cichlid historical biogeography has become so entrenched that the rifting history of Gondwana is routinely used to calibrate teleost molecular clocks [3,6], with the consequence that this hypothetical scenario now directly influences estimates of the evolutionary time scale for more than half of all modern vertebrate diversity.

If the distribution of modern cichlids is attributable purely to Gondwanan break-up, then it necessarily follows that the common ancestor of all living cichlids originated no later than the final separation between Madagascar–India and South America–Africa–Arabia. Current geological evidence places this continental fragmentation event in the Early Cretaceous (*ca* 135 Ma) [11]. However, the stratigraphically oldest fossil cichlids are Eocene in age (approx. 46 Ma) [12,13], implying a gap of approximately 90 Myr in the early history of the group. This, along with the absence of Early Cretaceous fossils belonging to more inclusive and taxonomically diverse clades that contain cichlids, has led some to abandon the orthodoxy of Mesozoic vicariance in favour of Cenozoic dispersal to explain the present-day distribution of cichlids [12,14].

The complete absence of fossil cichlids from many former Gondwanan landmasses would seem equally problematic for the vicariance hypothesis, but has received surprisingly little attention. For example, the Australian fossil record contains several fish-bearing freshwater deposits of Mesozoic and Cenozoic age, but no fossil cichlid is known from the continent. While it is clear that assembly of the compositionally distinctive Australian freshwater fish fauna has a complex history stemming from isolation, aridification and marine invasion coupled with the persistence of ancient lineages [15], this complexity does not undermine the prediction of the vicariance model that cichlids should have been widely distributed across Gondwanan landmasses during the Mesozoic [9].

These palaeontological arguments have been dismissed as ‘non-evidence’ by advocates of cichlid vicariance [16]. Some authors have even suggested that the fossil record supports the notion of cichlids deep within the Mesozoic, citing the high probability of non-preservation for freshwater taxa of Cretaceous age [9] or inferring that the advanced morphology of the earliest fossil cichlids implies a long, and as yet unsampled, palaeontological history of the group [9,10,13]. The seemingly ambiguous signal of palaeontological data with respect to the question of cichlid origin is symptomatic of a qualitative approach to an inherently quantitative problem. Invoked stratigraphic gaps are neither *ad hoc* contrivances nor trivial inconveniences to be dismissed as non-evidence; they are hypotheses amenable to statistical interrogation.

In order to provide a robust time scale for cichlid diversification and select between competing biogeographic hypotheses, we applied three semi-independent approaches in estimating the age of crown-group Cichlidae. Our first two methods are palaeontological, and draw on (i) the distribution of fossil horizons yielding cichlids and those that might plausibly yield cichlids (i.e. fish-bearing freshwater deposits on former Gondwanan landmasses) [17], and (ii) the stratigraphic distribution of more inclusive teleost lineages (meaning clades of higher taxonomic rank) that contain cichlids [18]. These techniques relate directly to two contrasting arguments that emerge repeatedly in palaeontological debates concerning the chronology of cichlid evolution: either that the record of freshwater fishes generally, and cichlids specifically, is sufficiently poor that the absence of Mesozoic cichlid fossils is unsurprising, or that the minimum age of origin for a series of more inclusive lineages of teleost fishes precludes the origin of cichlids deep within the Mesozoic. Significantly, these methods share only one common feature in their calculations: both are necessarily constrained by the minimum age for cichlids as imposed by the oldest fossil example(s) of the group. As an independent assessment of the divergence times estimated from palaeontological data, we conducted a relaxed-molecular-clock analysis for cichlids and Ovalentaria [19], a percomorph lineage that includes cichlids. Our dataset includes 10 protein-coding nuclear genes for 89 cichlids and 69 non-cichlid species of Percomorpha.

## 2. Material and methods

### (a) Estimating time of evolutionary origin using the distribution of cichlid-bearing fossil horizons

One method of estimating credible intervals (CIs) on stratigraphic durations draws on the number of fossil horizons within the sampled range of the group of interest. The simplest approach assumes that fossil horizons are distributed at random [20,21], but the potential for fossil recovery undoubtedly varies over time as a consequence of a heterogeneous rock record. Marshall [17] developed a more general method that permits non-uniform preservation by using an empirically informed function that quantifies potential for fossil recovery. We have applied this logic in conjunction with a Bayesian approach that provides a statistically appropriate framework for discussing the probability of clade origin within certain stratigraphic intervals [20]. Our results are conditioned on the prior assumption that cichlids are post-Palaeozoic in age (i.e. they originated in the Triassic or later), which is consistent with the fossil record and does not exclude the possibility of Gondwanan vicariance.

We assembled a database of known fossil occurrences of cichlids on Gondwanan landmasses based on the literature (see electronic supplementary material). Different geological formations (or localities where there is no formalized lithostratigraphic framework) were assumed to represent distinct sampling horizons. The function for the potential recovery of fossil cichlids was estimated by tabulating the number of sedimentary horizons (formations or localities) that meet three key criteria. First, candidate deposits must be present on former Gondwanan landmasses. Second, candidate deposits must represent freshwater environments. Third, candidate deposits must have the potential to yield fossils of cichlids, were this group present. Sites yielding fish fossils (including but not restricted to cichlids) meet this final criterion. The nature of fossils (articulated or fragmentary) from sites satisfying these conditions was also recorded.

Because of uncertainty surrounding age assessments, uniform recovery potential was assumed within each epoch-level stratigraphic bin, with relative recovery potential given by the number of candidate horizons present in a given interval divided by its duration. Ambiguity surrounds the age of many freshwater deposits. In this study, imprecisely dated deposits are given their oldest plausible age. This approach systematically biases analysis towards older age estimates for the time of clade origin, thereby providing a more generous test of the Gondwanan vicariance hypothesis.

These data were used to generate point estimates and 95% CIs for cichlid origin based on (i) the fossil records of Gondwanan landmasses currently inhabited by cichlids (South and Central America including the Caribbean, Africa, Madagascar, India, Arabia; cichlid fossils are known from all of these regions except Madagascar and India), and (ii) these records combined with those of Australia and Antarctica, former Gondwanan landmasses that lack cichlids but would be predicted to have once been inhabited by the group under the vicariance hypothesis. For both, we calculated CIs based on the record of all cichlid fossils and estimated range extensions based on articulated cichlid remains alone combined with appropriate recovery potential functions generated from the subset of deposits that yield complete fish specimens. This modified procedure is more conservative and reflects the very real possibility that the earliest cichlids might be recognized only on the basis of articulated remains, as their isolated fragments might be too character-poor, too generalized or both to permit reliable taxonomic attributions.

### (b) Estimating time of evolutionary origin using the distribution of ages of outgroups to cichlids

Hedman [18] devised a Bayesian approach for constraining the time of origin of a clade based on the distribution of stratigraphic ages of successive outgroups. This method requires that outgroups appear in the fossil record in an order matching phylogeny and that they pre-date or are contemporary with the first appearance of the focal clade. Such perfect congruence is rare in empirical examples, and we adopt a proposed solution that conservatively excludes inconsistent ages.

An account of the outgroups used in analysis is provided in the electronic supplementary material. In some cases, there is disagreement surrounding the identity of the earliest representatives of these lineages. To accommodate uncertainty, two sets of calculations were completed: one using the oldest proposed minimum age for a clade and the other applying the youngest. Collectively, these paired analyses provide upper and lower estimates of CIs for divergence times given present understanding of both the fossil record and teleost interrelationships. These age estimates are conditioned on a prior assumption that divergence occurred after a user-specified hard upper bound. This bound applies to the divergences of all groups considered, not only the focal clade. We have therefore selected the Carboniferous (Serpukhovian) *Discoserra*, a putative stem neopterygian [22], as defining an upper bound of 322.8 Ma (see the electronic supplementary material).

### (c) Collection of sequence data, phylogenetic analyses and relaxed molecular clocks

Standard phenol–chloroform extraction protocol or Qiagen DNeasy Blood and Tissue kits were used to isolate DNA from tissue biopsies sampled from 158 species of percomorph teleosts that included 89 species of Cichlidae (electronic supplementary material, table S6). Previously published PCR primers (see the electronic supplementary material) were used to amplify and sequence exons from 10 nuclear genes (*ENC1*, *Glyt*, *myh6*, *plagl2*, *Ptr*, *rag1*, *SH3PX3*, *sreb2*, *tbr1* and *zic1*). Amplified gene copies were cleaned and used as templates for DNA cycle sequencing. Alignments of the DNA sequences from the individual genes were constructed from the inferred amino acid sequences. Thirty data partitions were designated that corresponded to the three separate codon positions for each of the 10 protein-coding genes. A phylogeny of the aligned DNA matrix was inferred using maximum-likelihood and relaxed-clock analyses using a random local molecular-clock model in the computer program BEAST v. 1.6 (figure 1; electronic supplementary material, figure S1) [23,24]. DNA sequences are deposited on GenBank KF556709–KF557487. Aligned gene sequences used in phylogenetic analyses, phylogenetic trees resulting from RAxML and BEAST analyses, files formatted for BEAST analyses and files used to estimate the age of cichlids using palaeontological data are available from the dryad digital repository (http://dx.doi.org/10.5061/dryad.48f62). Fossil-based age constraints were applied to 10 nodes in the percomorph phylogeny (electronic supplementary material, figure S2).

### (d) Fossil calibration age priors

For each fossil calibration prior, we identify the calibrated node in the percomorph phylogeny, list the taxa that represent the first occurrence of the lineage in the fossil record, describe the character states that justify the phylogenetic placement of the fossil taxon, provide information on the stratigraphy of the rock formation(s) bearing the fossil, give the absolute age estimate for the fossil, outline the prior age setting in the BEAST relaxed-clock analysis and provide any additional notes on the calibration [25]. Each calibration is numbered and the phylogenetic placement of the calibration is highlighted in the electronic supplementary material, figure S3. Full justification of our calibrations is given in the electronic supplementary material.

Because we look to provide a critical test of competing models of cichlid biogeography, we have not assumed Gondwanan vicariance *a priori* and did not use the timing of the fragmentation history of this supercontinent to inform calibrations in the relaxed-molecular-clock analyses. Furthermore, we have not included any internal calibrations within Cichlidae, so that our relaxed-molecular-clock estimate of the evolutionary time scale for the group is truly independent of its fossil record, which contributes to our palaeontological estimates of divergence times (see §2).

## 3. Results

Our three approaches to estimating a time scale of cichlid origin and diversification yield overlapping CIs that diverge significantly from the predictions made by the Gondwanan vicariance biogeographic hypothesis, and are discussed in turn in §3*a*,*b* (figure 1).

### (a) Palaeontological time scales for cichlid evolution

The distribution of cichlid-bearing fossil horizons, combined with an empirically informed function describing fossil recovery potential, indicates an age of origin for cichlids in the Late Cretaceous or Palaeocene. If only the records of landmasses that are currently inhabited by cichlids are considered, the time of origin of the clade is estimated as 59.2 Ma (95% CI: 56.1–67.6 Ma). By contrast, a slightly younger age estimate of 57.8 Ma (95% CI: 56.1–62.4 Ma) is obtained if the record of all Gondwanan landmasses is considered. Restricting the scope of analysis to consider articulated remains alone provides a more conservative means of estimating the time of origin for cichlids, because early members of this group might not be recognized on the basis of less diagnostic skeletal debris. Point estimates for the timing of cichlid origin under this approach do not change drastically from those obtained using the entirety of the cichlid fossil record, but the upper bounds of the CIs do increase by more than 10 Ma. Depending on the scope of geographical analysis, we estimate the time of cichlid origin based only on articulated remains as ranging from 59.8 Ma (95% CI: 56.1–75.1 Ma; landmasses inhabited by modern cichlids) to 60.2 Ma (95% CI: 56.1–77.8 Ma; all Gondwanan landmasses). The Gondwanan vicariance hypothesis requires a pre-Eocene record of cichlids that is roughly 10–30 times worse than their recorded fossil history, with rescaled recovery potentials conditioned on point estimates for the origin of the group at 135 Ma ranging from 2.8–3.3% (all fossils) to 6.6–6.9% (articulated fossils only) of their original values. Classical confidence intervals deliver similar results to the Bayesian estimates (see electronic supplementary material, table S2).

Analysis of outgroup ages provides broadly similar estimates for the timing of cichlid origin to those derived from the distribution of cichlid fossil horizons, in terms of both the magnitude of point estimates and the degree of uncertainty surrounding them. We find a mean age of 60.7 Ma (95% CI: 46.8, 90.1 Ma) using the oldest possible fossil ages for outgroups. The time scale for cichlid origin is predictably more recent using the youngest possible fossil ages for outgroups, but only slightly so, with a mean age of 57.0 Ma (95% CI: 46.8–81.2 Ma). Using this same approach, it is also possible to determine probable times of origin for a series of more inclusive clades that contain Cichlidae: Ovalentaria, Percomorpha, Acanthopterygii, Acanthomorpha, Eurypterygii, Euteleostei and Teleostei. This exercise implies that no crown acanthomorph lineages are likely to be sufficiently ancient to have vicariant Gondwanan distributions, as we estimate the age of the group as between 106.4 Ma (95% CI: 98.5–132.2 Ma) and 109.2 Ma (95% CI: 98.5–136.0 Ma). The most restrictive group containing cichlids that we can date with this method and which is of sufficient apparent antiquity to have been affected by the initial rifting of Gondwana is Eurypterygii, the radiation containing Acanthomorpha, Myctophiformes and Aulopiformes [25]. Our estimates for the time of origin for this major teleost clade range between 131.1 Ma (95% CI: 104.9–163.2 Ma) and 142.1 Ma (95% CI: 126.2–166.2 Ma).

### (b) A molecular time scale for cichlid evolution

The phylogeny of Ovalentaria and the major cichlid lineages inferred from the 10 nuclear genes is similar to previous molecular and morphological analyses [8,10,19], with Etroplinae (India, Madagascar) resolved as the earliest-diverging clade and Ptychochrominae (Madagascar) as the sister lineage to the unnamed clade that contains the African (Pseudocrenilabrinae) and Neotropical (Cichlinae) cichlid lineages (figure 1; electronic supplementary material, figure S1). The 10 nuclear gene phylogeny preserves the parallels between patterns of cichlid interrelationships and the fragmentation history of Gondwana that has led to the prominence of vicariance biogeographic scenarios for this lineage [9]. However, the Bayesian random local molecular-clock analyses yield age estimates for the origin of cichlids consistent with those derived from analysis of fossils alone (figure 1 and table 1; electronic supplementary material, figure S1).

**Table 1.** Posterior molecular age estimates for major lineages of Cichlidae. Ages refer to crown groups.

clade	mean age (Ma)	95% highest posterior density interval (Ma)
Cichlidae	64.9	57.3–76.0
Etroplinae (India and Madagascar)	36.0	30.3–42.2
Ptychochrominae (Madagascar)	38.2	31.7–46.4
unnamed Afro-American clade	46.4	40.9–54.9
Cichlinae (neotropics)	29.2	25.5–34.8
Pseudocrenilabrinae (Africa)	43.7	38.2–51.6
unnamed east African clade	8.0	6.9–9.5
most recent common ancestor of Lake Malawi and Lake Victoria radiations	2.3	1.7–3.1
Crater Lake Barombi Mbo (Cameroon)	1.4	0.8–2.3

Based on the timing of Gondwanan fragmentation events, crown cichlids should occur in the Early Cretaceous or Late Jurassic [3,6,9,10]; however, the Bayesian random local molecular-clock analyses place the origin of the modern cichlid radiation near the Cretaceous–Palaeogene boundary (figure 1 and table 1; electronic supplementary material, figure S1), with a mean age estimate of 64.9 Ma (95% CI: 57.3–76.0 Ma). The estimated age of the most recent common ancestor (MRCA) of cichlids and their sister lineage, *Pholidichthys*, is also younger (mean: 103.7 Ma; 95% CI: 92.0–118.4 Ma) than the initial rifting of Gondwana at approximately 135 Ma [11]. The mean estimated age of the MRCA of the African and Neotropical cichlids was 46.4 Ma (95% CI: 40.9–54.9 Ma), post-dating the final separation of Africa and South America by more than 40 Myr. The cichlid time tree confirms ages estimated in previous studies for the east African [26] (mean: 8.0 Ma; 95% CI: 6.9–9.5 Ma) and Cameroon crater lake Barombi Mbo [27] radiations (mean: 1.4 Ma; 95% CI: 0.8–2.3 Ma), verifying relatively young ages for these remarkable examples of adaptive radiation (figure 1 and table 1; electronic supplementary material, figure S2). The age estimate in the 10 nuclear gene inferred time tree closest to the timing of Gondwanan fragmentation is that of the inclusive (mean: 123.5 Ma; 95% CI: 111.4–136.2 Ma), but unnamed, percomorph clade that contains more than one-quarter of all living vertebrate species (approx. 16 570 species), including cichlids (see electronic supplementary material, figure S2).

## 4. Discussion

### (a) Congruence between palaeontological and molecular time scales for cichlid evolution

The application of two contrasting palaeontological approaches in calculating temporal range extensions yields strikingly congruent time scales for cichlid evolution. Both methods provide point estimates for the origin of the group that range between 57 and 60 Ma (Palaeocene), and strongly reject the possibility that crown cichlids are sufficiently old to have been affected by the initial rifting of Gondwana. Instead, upper limits for the origin of cichlids lie consistently within the late Late Cretaceous. This congruence is particularly compelling because the methods that yielded these comparable results share only one similarity in their calculations: both are constrained by the minimum age for cichlids as imposed by the oldest fossil example of the group.

Our molecular time tree provides a mean estimate for the timing of cichlid origin in the Palaeocene, but cannot reject the possibility that the group arose as early as the Late Cretaceous. This result is consistent with other recent molecular-clock estimates for the origin of cichlids that do not assume Gondwanan vicariance for the group *a priori*, and which range in age from Late Cretaceous to Eocene [28–33]. In terms of point estimates and surrounding uncertainty, our revised molecular time scale is entirely consistent with the ages derived from analyses of the fossil record alone (figure 1). It is important to note that our relaxed-molecular-clock analysis shares no palaeontological data in common with either our analysis of the distribution of cichlid-bearing fossil horizons or our database of outgroup-based age constraints. We interpret the convergence of these three semi-independent approaches, which all deliver age estimates for cichlids that are within error of one another, as a consequence of genuine evolutionary signal that strongly contradicts the time scales for cichlids demanded by hypotheses of Gondwanan vicariance.

### (b) The timing of cichlid diversification: congruence and incongruence

Our estimates for the time of cichlid origin are congruent not only with one another, but also with previous molecular time scales for the evolution of this group that do not assume a Gondwanan vicariance scenario *a priori* [4,5,28–33]. The oldest such estimates from previous work are early Late Cretaceous [3], pre-dating our proposed time of origin by roughly 35–45 Myr. However, these more ancient dates derived from analysis of mitochondrial sequences, which are characterized by high rates of nucleotide substitution that might bias clock analyses towards older estimated times of divergence [25,34–36].

Generally, the only molecular-clock analyses to deliver time scales consistent with the predictions of the vicariance hypothesis were themselves calibrated using a combination of age constraints from the fossil record and Gondwanan fragmentation events [3,5,6,37]. There is no published relaxed-molecular-clock analysis that results in an Early Cretaceous or Jurassic origin of cichlids that is independent of the ages implied by the timing of the fragmentation of western Gondwana.

Our palaeontological time scales for Cichlidae constrain only the origin of the group, but our time-calibrated phylogeny permits investigation of the timing of deep divergences within the clade (table 1). We estimate the divergence of South American and African cichlids as Eocene, with the origin of the African cichlid crown within the same interval. This is consistent with the placement of the middle Eocene (approx. 46 Ma) †*Mahengechromis* as an early crown pseudocrenilabrine [12]. By contrast, our estimated Eocene–Oligocene age for the South American cichlid crown contradicts published interpretations of the fossil cichlids from the ‘Faja Verde’ level of the Lumbrera Formation of Argentina. These fossils are often cited as early–middle Eocene in age [13,38,39], leading to calibration minima of 49 Ma in recent molecular clock studies [6]. However, the hard minimum for the age of these fossils is 33.9 Ma, which derives from radiometric dating of overlying tuff layers [40]. This more appropriate minimum age estimate only partially reconciles our time scale with previous phylogenetic interpretations of the Lumbrera cichlids, each of which has been placed within the South American crown in association with specific cichline tribes (†*Protocara* as either a geophagine or a stem member of an unnamed clade comprising Chaetobranchini, Geophagini, Cichlasomatini and Heroini [39,41]; †*Gymnogeophagus eocenicus* as phylogenetically nested within a living genus [38]; and †*Plesioheros* as a crown heroine [13]). It is difficult to evaluate confidence in the evolutionary relationships proposed for these fossils because published analyses using morphological data do not provide support for nodes in accompanying phylogenies (e.g. bootstrap resampling scores or Bayesian posterior probabilities). We also note that some phylogenetic hypotheses derive from successive reweighting exercises [39], while others assume restricted placement of fossil species prior to analysis [13]. There is no doubt that Lumbrera cichlids are significant on account of their antiquity and geographical provenance. However, in the absence of demonstrably robust phylogenetic placements of these fossil lineages within a group well known for convergent morphological evolution [42], their exact implications for the timing of major events in cichlid evolution are likely to remain ambiguous.

### (c) Comparison with other putative examples of Gondwanan vicariance

Among vertebrates assumed to have limited dispersal ability across marine barriers, cichlids are not unique in showing a broad distribution across southern landmasses combined with a fossil record that commences long after the tectonic break-up of Gondwana. Several groups of freshwater fishes, reptiles, mammals and plants show disjunct distributions, with members present in South America and Africa, but only a few instances seem definitively attributable to drift-based vicariance [14,43,44]. Instead, molecular clock analyses for a range of groups with apparent vicariant distributions across southern continents [45–48] paint a picture of widespread ‘pseudo-congruence’, where similar biogeographic patterns originate at different times that may be disjunct with the age of specific palaeogeographic events [49].

Our consistent time scales for cichlid evolution reject Gondwanan vicariance as a viable mechanism for the modern distribution of the group, but they demand what can only be considered a series of highly unlikely transoceanic dispersal events. Like the fossil record, the salinity tolerance of cichlids has been subjected to contrasting interpretations; it has been cited as both consistent [12] and inconsistent [10] with marine dispersal. Experimental evidence points to high salinity tolerance in some cichlids [50,51], but the fact that no cichlid inhabits the open ocean indicates that long-distance marine migration is improbable. Dispersal across the south Atlantic would appear to be especially unlikely, given that it measured roughly 1000 km [52] in width by the time of the inferred divergence between South American and African cichlids in the Eocene (figure 1). Despite the presence of a substantial marine barrier, it is clear that at least two groups of terrestrial mammals—primates and hystricognath rodents—dispersed from Africa to South America at approximately this time [53]. More generally, there is strong evidence from other animal groups and plants for surprisingly high levels of biotic interchange between South America and Africa throughout the Late Cretaceous and Palaeogene [54,55]. Geological evidence indicates the presence of a chain of now-submerged islands across the south Atlantic during the Palaeogene [52]. These islands coincided with strong east-to-west palaeocurrents across the south Atlantic and both have been invoked as key elements of a selective dispersal route from Africa to South America during the Eocene [12,52]. It is also possible that freshwater plumes, such as that produced by the modern Congo River [56], provided corridors of brackish surface water that could have permitted migration by freshwater taxa across a narrower marine barrier during the Palaeogene.

Coeval examples of migration provide circumstantial evidence for the possibility of trans-Atlantic dispersal, and geographical factors during the Eocene would appear to have facilitated the crossing, but cichlid migration across the south Atlantic and other marine barriers nevertheless remains an extraordinary claim. However, the evolutionary time scale inferred for cichlids on the basis of both fossils and molecules demands that this hypothesis is given serious consideration rather than being dismissed *a priori*.

Our estimation of consistent palaeontological and molecular ages for the origin of cichlids adds to a growing number of studies reporting close congruence between divergence time estimates from ‘rocks’ and ‘clocks’, in cases where these approaches had previously delivered wildly different evolutionary time scales [57]. This convergence would seem to signal the end of an era dominated by debates on the relative merits of molecular and fossil data, permitting molecular biologists and palaeontologists to move forward on addressing questions related to the timing of major events underpinning the origin of modern biodiversity.
